# Microvascular Failure in the Aging Brain: Converging Pathways of Oxidative Stress, Inflammation, and Endothelial Decline

**DOI:** 10.3390/biomedicines14010130

**Published:** 2026-01-08

**Authors:** Jordana Mariane Neyra Chauca, Maclovia Vázquez VanDyck, Armando Espinoza Santana, Graciela Gaddy Robles Martínez, Kalid Alejandra Romero Vega, Nancy García Quintana, Vanessa Favila Sánchez

**Affiliations:** 1 Facultad de Medicina, Universidad Autónoma de Guadalajara, Guadalajara 45129, Mexico; maclovia.vazquez@edu.uag.mx (M.V.V.); drachela87@gmail.com (G.G.R.M.); kalid.romero@edu.uag.mx (K.A.R.V.); 2 Facultad de Medicina, Benemérita Universidad Autónoma de Puebla, Puebla 72420, Mexico; armandoespinozasantana@gmail.com; 3 Facultad de Medicina, Universidad Autónoma del Estado de México, Toluca 50180, Mexico; garciaquintananancy@yahoo.com.mx (N.G.Q.); vane_favila@outlook.com (V.F.S.)

**Keywords:** microcirculation, oxidative stress, endothelial dysfunction, inflammation, peripheral artery disease, blood–brain barrier, vascular aging, neurovascular unit, microthrombosis

## Abstract

**Background**: Aging exerts a progressive and multifaceted impact on the microcirculatory system, undermining the structural and molecular integrity that sustains endothelial stability across both peripheral and cerebral vascular territories. A sustained shift toward oxidative imbalance, chronic low-grade inflammation, and progressive endothelial exhaustion converges to destabilize microvascular networks, linking peripheral artery disease (PAD) with heightened susceptibility to cerebral microvascular dysfunction and neurovascular decline. As redox homeostasis deteriorates, endothelial cells progressively lose barrier-selective properties, intercellular communication with pericytes weakens, and pro-thrombotic tendencies subtly emerge, creating a permissive environment for early neurovascular injury and impaired cerebrovascular resilience. **Methods**: This narrative review integrates mechanistic evidence derived from experimental, clinical, and translational studies examining the interplay between oxidative stress, inflammatory signaling cascades, endothelial senescence, and blood–brain barrier (BBB) disruption across peripheral and cerebral microvascular systems. A comparative framework was applied to PAD and cerebral microcirculatory pathology to identify convergent molecular drivers and systemic mechanisms underlying endothelial deterioration. **Results**: Accumulating evidence demonstrates that oxidative stress disrupts endothelial mitochondrial function, compromises tight junction architecture, and accelerates angiogenic failure. Concurrent inflammatory activation amplifies these alterations through cytokine-mediated endothelial activation, enhanced leukocyte adhesion, and promotion of a pro-thrombotic microenvironment. Progressive endothelial senescence consolidates these insults into a persistent state of microvascular dysfunction characterized by diminished nitric oxide bioavailability, capillary rarefaction, and compromised barrier integrity. Notably, these pathological features are shared between PAD and the aging cerebral circulation, reinforcing the concept of a unified systemic microvascular aging phenotype. **Conclusions**: Microvascular failure in the aging brain should be understood as an extension of systemic endothelial deterioration driven by oxidative stress, chronic inflammation, and senescence-associated vascular exhaustion. Recognizing the shared molecular architecture linking peripheral and cerebral microcirculatory dysfunction offers a strategic framework for developing targeted therapeutic interventions aimed at restoring endothelial resilience, stabilizing BBB integrity, and preserving neurovascular homeostasis in aging populations.

## 1. Introduction

As the microcirculation becomes increasingly vulnerable to metabolic and immunological pressures, a subtle yet consequential restructuring of the vascular environment takes place. The endothelial surface—once a dynamic interface capable of sensing shear forces, regulating coagulation, and preserving molecular selectivity at the blood–tissue barrier—progressively loses its adaptive capacity. This deterioration reflects the cumulative burden of oxidative imbalance, chronic inflammatory signaling, and hemodynamic instability that accumulate over decades in both physiological aging and vascular disease [1,2]. In peripheral artery disease (PAD), circulating microparticles released from activated or apoptotic endothelial cells have emerged as sensitive biomarkers of this vascular attrition [3]. Notably, similar circulating patterns have been detected in individuals with early cognitive impairment, reinforcing the concept that microvascular degeneration—whether peripheral or cerebral—is embedded within a broader systemic vascular phenotype [4].

This evolving perspective challenges the traditional compartmentalization of vascular pathology. PAD has historically been regarded as a disease confined to the lower extremities, whereas neurodegenerative and cerebrovascular disorders were considered exclusive to the central nervous system. However, accumulating evidence indicates that the microcirculation functions as a unified vascular network whose integrity depends on coordinated biochemical and immune homeostasis [1,5]. Disturbances originating in the periphery propagate through systemic circulation, modifying endothelial gene expression, redox balance, and inflammatory thresholds in remote vascular beds [6]. These systemic perturbations help explain the elevated incidence of stroke, cognitive decline, and accelerated brain aging observed in patients with PAD—phenomena that cannot be fully accounted for by traditional cardiovascular risk factors alone [4].

At the mechanistic level, oxidative stress emerges as a central mediator of this vascular continuum. Mitochondrial dysfunction within endothelial cells drives excessive production of superoxide and hydrogen peroxide, initiating a self-amplifying cycle in which redox imbalance disrupts nitric oxide signaling and impairs vasodilatory responses [7]. Concurrently, oxidation of membrane lipids and cytoskeletal proteins destabilizes endothelial junctional complexes and compromises barrier integrity [8]. These molecular disruptions mirror those observed in cerebral microvessels exposed to chronic metabolic stress, where ROS-driven degradation of tight junction architecture contributes to early blood–brain barrier (BBB) permeability and altered neurovascular coupling [9].

Inflammatory processes further intensify these oxidative alterations, transforming physiologic immune surveillance into a sustained vascular threat. The aging immune system becomes increasingly prone to dysregulated cytokine production, with interleukin-6, tumor necrosis factor-α, and C-reactive protein exerting profound modulatory effects on endothelial behavior [10]. In PAD, this inflammatory milieu promotes leukocyte–endothelial interactions, platelet adhesion, and microthrombus formation in distal capillary beds [3,11]. Analogous events have been documented in the cerebral microcirculation, where inflammatory upregulation leads to capillary constriction, impaired functional hyperemia, and disruption of perfusion dynamics [12]. These parallel inflammatory pathways highlight a shared vulnerability linking peripheral and cerebral microvascular territories.

A critical driver of progressive microvascular decline is the onset of endothelial senescence. Triggered by sustained oxidative and inflammatory stress, senescent endothelial cells adopt a dysfunctional phenotype marked by reduced proliferative capacity, altered metabolic function, and secretion of a senescence-associated secretory profile (SASP) enriched in pro-inflammatory and matrix-remodeling factors [13]. As these cells accumulate, they distort local hemodynamics, diminish angiogenic potential, and degrade extracellular matrix integrity. In both PAD and the aging brain, endothelial senescence erodes vascular adaptability, rendering the microcirculation increasingly susceptible to occlusion, hypoperfusion, and barrier breakdown [5,14].

Recognizing these shared mechanisms carries critical implications for diagnosis and therapy. Approaches that isolate PAD and neurovascular disorders as separate entities risk overlooking the systemic drivers that orchestrate disease evolution. By reframing microvascular pathology as a unified, multi-territory process, therapeutic strategies can be directed toward restoring redox equilibrium, attenuating inflammation, stabilizing endothelial function, and reinforcing BBB integrity [7,10,15].

The present review integrates these perspectives, situating microvascular failure within a broader pathophysiological continuum linking peripheral artery disease to cerebral microcirculatory impairment. By examining the interconnected roles of oxidative stress, inflammation, endothelial senescence, and BBB dysfunction, we propose a unified model of systemic vascular aging that underpins both peripheral and central vascular pathology [1,4,5,9].

This review goes beyond descriptive synthesis by proposing an integrated mechanistic framework in which peripheral artery disease and cerebral microvascular dysfunction are redefined as interconnected manifestations of systemic vascular aging. By positioning oxidative stress, chronic inflammation, endothelial senescence, and neurovascular unit disruption as convergent drivers, this work offers a unified conceptual model that helps reinterpret peripheral vascular disease as an early sentinel of cerebral vulnerability and cognitive decline [1,3,4,5,6,9].

## 2. Methods

This manuscript was developed as a narrative review following the Scale for the Assessment of Narrativo Review Articles (SANRA) framework to ensure methodological rigor, transparency, and coherence. The review was guided by a clearly defined research focus addressing the role of microvascular dysfunction in aging, emphasizing oxidative stress, chronic inflammation, endothelial senescence, and blood–brain barrier (BBB) disruption as shared molecular drivers linking peripheral artery disease (PAD) and cerebral microvascular decline.

A structured literature search was conducted in PubMed/MEDLINE, Scopus, Web of Science, and Google Scholar for publications between 2000 and 2025. Search terms included: “microvascular dysfunction”, “vascular aging”, “oxidative stress”, “endothelial senescence”, “blood–brain barrier”, “neurovascular unit”, “inflammation” and “peripheral artery disease”. Reference lists of key articles were also screened to ensure comprehensive coverage.

Articles were selected based on their scientific relevance, conceptual contribution, and methodological quality, prioritizing peer-reviewed experimental, clinical, and translational studies. Data were interpreted through a thematic narrative synthesis organized into mechanistic domains, ensuring logical structure, thematic coherence, and balanced discussion in accordance with SANRA criteria.

Although not a systematic review, this approach provides a structured and reproducible synthesis aligned with recognized standards for narrative reviews.

### 2.1. Quality Assessment and Risk of Bias Evaluation

Although this manuscript was developed as a narrative review, a structured quality appraisal was incorporated to enhance methodological transparency, following the editor’s recommendation. We therefore applied the ROBIS (Risk of Bias in Systematic Reviews) framework in an adapted qualitative manner to evaluate the reliability and interpretability of the evidence included.

The appraisal began with an assessment of study relevance, ensuring that each selected article contributed meaningfully to the mechanistic domains addressed in this review, including oxidative stress, inflammation, endothelial senescence, microvascular dysfunction, and blood–brain barrier (BBB) impairment. Studies lacking conceptual alignment or presenting insufficient methodological description were excluded from the synthesis.

The ROBIS tool was then used to qualitatively evaluate four core domains: (1) study selection and identification, (2) data collection and appraisal, (3) synthesis and interpretation, and (4) overall relevance. Each domain was reviewed for potential concerns affecting the validity of mechanistic conclusions, with judgments classified as low, unclear, or high concern. This evaluation was integrated into the narrative interpretation rather than applied as an exclusion criterion, as is appropriate for narrative reviews.

Overall, most studies demonstrated a low to moderate concern for bias, with the highest issues observed in incomplete reporting of experimental methodology and variability in preclinical design quality. These considerations were incorporated into the discussion to ensure a transparent interpretation of the strength and limitations of the available evidence.

### 2.2. Evidence Source Distribution

The mechanistic evidence synthesized in this review originates from multiple experimental domains. Approximately 45–50% of studies derive from animal models, including murine models of endothelial senescence, oxidative stress, and BBB dysfunction [16,17,18]. Around 25–30% correspond to in vitro experiments, particularly endothelial cell cultures, pericyte–endothelial co-culture systems, and BBB-on-chip platforms that enable controlled molecular interrogation [19,20,21]. Finally, 20–25% of the evidence comes from human clinical or translational studies, including circulating inflammatory biomarkers [18,22], oxidative stress markers [1,3], endothelial microparticles [23], and neuroimaging measures of BBB leakage and perfusion deficits [24,25,26]. This distribution highlights the need for expanded human research to validate the mechanistic pathways identified in preclinical models.

## 3. Oxidative Stress in Peripheral and Cerebral Microcirculation

Oxidative stress represents one of the earliest and most pervasive pathological drivers of microvascular dysfunction across both peripheral and cerebral vascular territories. Although traditionally framed as an imbalance between reactive oxygen species (ROS) generation and antioxidant defenses, oxidative stress within the microvasculature constitutes a multidimensional disruption encompassing mitochondrial bioenergetics, redox-sensitive signaling pathways, endothelial metabolic regulation, and structural stability of the vascular wall [2,6,22]. In both peripheral artery disease (PAD) and the aging brain, sustained accumulation of ROS and reactive nitrogen species progressively erodes endothelial homeostasis, initiating a cascade that culminates in microvascular instability and functional decline [1,5].

In the peripheral circulation, particularly in PAD, oxidative stress operates simultaneously as both a causal mechanism and a consequence of chronic ischemia. Reduced perfusion compromises mitochondrial oxidative phosphorylation within endothelial cells, promoting excessive superoxide generation, mitochondrial fragmentation, and bioenergetic failure [2,21,22]. Concurrently, activation of NADPH oxidase isoforms—primarily NOX2 and NOX4—amplifies ROS production, overwhelming intrinsic antioxidant systems such as superoxide dismutase and glutathione peroxidase [6,27]. This oxidative overload alters endothelial phenotype at multiple levels, including diminished nitric oxide (NO) bioavailability, lipid peroxidation of cellular membranes, cytoskeletal disorganization, and impaired modulation of barrier permeability, ultimately compromising microvascular adaptability and perfusion regulation [5,10,15].

Importantly, oxidative perturbations in PAD are not confined to the ischemic limb. Circulating oxidized lipoproteins, lipid peroxidation products, and redox-modified proteins propagate systemic vascular injury and modify endothelial function in distant vascular beds, including the cerebral microcirculation [1,3]. Experimental and clinical data indicate that these oxidative byproducts destabilize tight junction architecture and impair cerebrovascular reactivity even in the absence of localized cerebral ischemia, reinforcing the concept of PAD as a systemic amplifier of microvascular pathology [3,28].

Within the cerebral microvasculature, oxidative stress follows a parallel yet metabolically specialized trajectory. Cerebral endothelial cells exhibit exceptionally high mitochondrial density to sustain the energetic demands of the blood–brain barrier (BBB) [29,30]. Age-related mitochondrial decline reduces ATP availability and disrupts calcium homeostasis, predisposing these cells to redox imbalance even under normoxic conditions [21,31]. The resulting overproduction of superoxide and peroxynitrite destabilizes tight junction complexes, alters cytoskeletal organization, and increases endothelial permeability, thereby compromising BBB integrity and neurovascular coupling [32,33].

Beyond the high mitochondrial density of cerebral endothelial cells, the brain exhibits additional sources of reactive oxygen species (ROS) that contribute to its uniquely oxidizing microenvironment. Microglial activation, NOX2-dependent enzymatic ROS production, and the high metabolic rate of neuronal tissue generate sustained oxidative pressure that is not observed in peripheral vascular beds [29,30,34]. Moreover, the lipid-rich composition of the brain increases susceptibility to peroxidation, further amplifying ROS generation during aging and inflammation [34,35]. To counterbalance this vulnerability, astrocytes provide critical antioxidant support through glutathione synthesis, ROS scavenging, metabolic buffering, and maintenance of redox homeostasis at the neurovascular interface [36,37,38]. These CNS-specific mechanisms emphasize the distinct oxidative environment of the brain and help explain why cerebral microvessels are particularly sensitive to oxidative injury compared with peripheral circulation.

One of the earliest structural manifestations of oxidative injury in the brain involves degradation and mislocalization of tight-junction proteins such as claudin-5 and occludin, events that precede overt microvascular breakdown [33,39]. This subtle disruption facilitates paracellular leakage of plasma-derived proteins, including fibrinogen and albumin, into the neural microenvironment, further propagating oxidative and inflammatory cascades that reinforce endothelial dysfunction [40,41].

Endothelial nitric oxide synthase (eNOS) occupies a critical central position within this oxidative pathology. Under physiological conditions, eNOS supports vasodilation and vascular protection; however, oxidative depletion of tetrahydrobiopterin (BH4) induces eNOS uncoupling, shifting its activity toward superoxide generation rather than NO synthesis [15,42]. This maladaptive transition, documented in both PAD and aging cerebral microvessels, contributes to impaired perfusion, endothelial paralysis, and progressive microvascular rarefaction [43,44].

Hemodynamic disturbances further exacerbate oxidative injury. Disturbed flow patterns and oscillatory shear stress in PAD activate redox-sensitive transcription factors such as NF-κB, enhancing oxidative enzyme expression while suppressing antioxidant defenses [45,46]. Similarly, in the aging cerebral circulation, arterial stiffness and altered pulsatility promote localized ROS accumulation and increased endothelial vulnerability [47]. These mechanical and biochemical stressors converge to accelerate endothelial apoptosis, extracellular matrix degradation, and capillary dropout [48,49].

Beyond structural alterations, oxidative stress promotes a prothrombotic transformation of the microvascular surface. Oxidative modification of endothelial membranes increases platelet adhesion, upregulates von Willebrand factor expression, and enhances tissue factor activation, facilitating microthrombus formation in both peripheral and cerebral microvascular beds [16,50].

Collectively, oxidative stress constitutes a unifying pathogenic mechanism linking PAD and cerebral microvascular degeneration. Its convergence across mitochondrial dysfunction, NADPH oxidase activation, lipid peroxidation, and impaired eNOS signaling underscores its central role in systemic microvascular aging. The peripheral manifestation of oxidative imbalance in PAD therefore represents an early sentinel of parallel vulnerabilities within the cerebral microcirculation [1,51].

Taken together, these processes highlight the need to integrate oxidative stress, chronic inflammation, and cellular senescence into a unified explanatory framework. Excess ROS generation activates redox-sensitive inflammatory pathways and cytokine cascades, particularly involving IL-6, TNF-α, and IL-1β [2,6,16,22,52,53]. Persistent inflammatory signaling further amplifies oxidative injury and endothelial dysfunction [9,17,52,53]. This pro-oxidant, pro-inflammatory environment promotes endothelial and glial senescence, driven by SASP-mediated secretion of cytokines, chemokines, and proteases [12,48,54,55,56,57]. Senescent endothelial cells and perivascular immune cells exacerbate ROS imbalance, sustaining a self-reinforcing “vicious cycle” of inflammatory and oxidative stress-driven microvascular aging [9,12,17,54]. Importantly, this bidirectional feedback loop provides a mechanistic link between peripheral vascular dysfunction in PAD and cerebral microvascular impairment observed in aging and neurodegenerative disease [1,3,22,25,28,33,35,51,58].

### Theoretical Framework: Vascular Aging as a Systemic Process

Vascular aging is increasingly recognized as a systemic phenomenon rather than a process limited to a single vascular bed. This perspective emerges from the convergence of three fundamental mechanisms—oxidative stress, chronic inflammation, and cellular senescence—which occur simultaneously across peripheral and cerebral microcirculations. Excessive production of reactive oxygen species (ROS) triggers endothelial injury, reduces nitric oxide (NO) bioavailability, and activates redox-sensitive inflammatory pathways such as NF-κB signaling [2,6,16,22,53,59]. Chronic vascular inflammation further amplifies endothelial dysfunction through sustained secretion of cytokines, including IL-1β, IL-6, and TNF-α, establishing a pro-inflammatory milieu that accelerates microvascular deterioration across organ systems [9,17,52,53,60,61].

This inflammatory environment promotes premature senescence of endothelial and perivascular cells, leading to a senescence-associated secretory phenotype (SASP) enriched with cytokines, chemokines, proteases, and ROS-amplifying mediators [12,48,54,55,56,57]. Senescent endothelial cells progressively lose functional specialization, impair vasoreactivity, and compromise barrier integrity, ultimately perpetuating a self-reinforcing cycle of oxidative stress and inflammation. These interconnected mechanisms contribute to systemic microvascular rarefaction, impaired perfusion, and endothelial decline observed in cardiovascular aging, peripheral artery disease, and cerebrovascular dysfunction [1,3,22,25,28,33,35,62].

**Figure 1:**
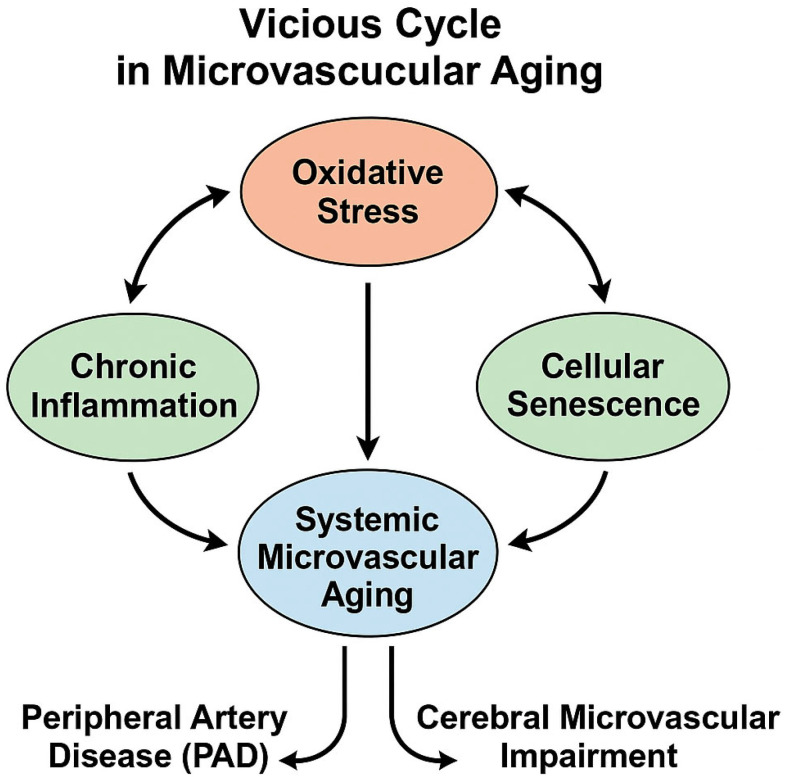
Integrated “vicious cycle” linking oxidative stress, chronic inflammation, and cellular senescence as mutually reinforcing drivers of systemic microvascular aging. These processes converge to impair both peripheral (PAD) and cerebral microcirculatory function.

In the brain, these systemic mechanisms intersect with neurovascular unit instability and blood–brain barrier (BBB) vulnerability, which share the same upstream triggers of oxidative stress, immune activation, and endothelial senescence [33,35,58]. Consequently, microvascular aging should be understood as an organism-wide decline in vascular resilience driven by shared molecular pathways that manifest in parallel across multiple tissues. This conceptual framework supports the view that cerebrovascular impairment in aging and Alzheimer’s disease represents a regional expression of global vascular aging. Figure 1 summarizes these bidirectional feedback loops. Key circulating biomarkers reflecting these shared microvascular pathways across peripheral artery disease and cerebral microvascular dysfunction are summarized in Table 1.

## 4. Chronic Inflammation and Microvascular Vulnerability

Chronic inflammation operates as a sustained and self-perpetuating force that progressively reconfigures microvascular physiology in both peripheral and cerebral territories, transcending its role as a secondary consequence of tissue injury and emerging as a central driver of vascular fragility and functional decline [52,53]. Unlike acute inflammation, which is tightly regulated and temporally constrained, chronic inflammatory activation evolves insidiously, fueled by metabolic dysregulation, immune senescence, endothelial stress, and age-associated shifts in redox balance [53,66]. This persistent low-grade inflammatory environment gradually erodes microvascular resilience, destabilizes barrier integrity, disrupts hemodynamic homeostasis, and establishes a permissive substrate for ischemia, hypoperfusion, and progressive structural degeneration [67]. In both peripheral artery disease (PAD) and the aging brain, chronic inflammation therefore functions as a pathophysiological nexus linking peripheral vascular impairment to cerebral microvascular vulnerability through a shared immunovascular continuum [52,59].

Both peripheral and cerebral microvascular beds exhibit convergent inflammatory signatures characterized by elevations of IL-6, TNF-α, and IL-1β. These cytokines are consistently increased in PAD, where they promote endothelial dysfunction, leukocyte adhesion, and redox dysregulation [18,22,52]. Similarly, in neurodegenerative disorders, these same pro-inflammatory mediators drive microglial activation, oxidative stress amplification, and endothelial destabilization [34,63]. A central regulatory node linking these processes is the NF-κB pathway, which is activated by oxidative cues in PAD and by chronic neuroinflammation in the aging brain, coordinating transcription of cytokines, adhesion molecules, and oxidative enzymes [53,60,61]. The shared elevation of these cytokines and the common reliance on NF-κB signaling underscore the molecular continuity between peripheral vascular inflammation and cerebrovascular vulnerability.

Within the peripheral circulation, chronic inflammation emerges early in the natural history of PAD and progressively evolves into a dominant force governing endothelial behavior. Sustained elevation of inflammatory cytokines and acute-phase mediators reflects the continuous activation of innate and adaptive immune pathways [23,53,66]. Far from being passive markers, these mediators actively transform endothelial phenotype, promoting a pro-adhesive, pro-thrombotic, and permeability-prone state. Recurrent cytokine signaling disrupts nitric oxide bioavailability, enhances vascular stiffness, and accelerates endothelial activation, reinforcing local ischemia and amplifying inflammatory signaling through a maladaptive feedback loop [16,18,23].

The ischemic environment characteristic of PAD further intensifies this inflammatory cascade. Hypoxia activates hypoxia-inducible factors, which orchestrate the upregulation of chemokines and cytokines responsible for leukocyte recruitment and vascular infiltration [67]. Recruited immune cells undergo phenotypic polarization toward pro-inflammatory states, releasing myeloperoxidase, reactive oxygen species, and proteolytic enzymes that degrade extracellular matrix components and damage the endothelial surface, thereby exacerbating microvascular instability [59]. These processes not only aggravate local tissue injury but also contribute to systemic dissemination of inflammatory mediators.

Crucially, PAD-associated inflammation is not spatially confined to the affected limb. Circulating cytokines and immune-derived factors exert distal effects on the cerebrovascular system, altering endothelial responsiveness and accelerating cerebral microvascular dysfunction [23,28]. Epidemiological and mechanistic evidence consistently demonstrates that individuals with PAD exhibit an increased burden of cognitive decline, ischemic events, and cerebral small-vessel disease, underscoring the systemic character of chronic vascular inflammation [13,28].

Within the central nervous system, chronic inflammation arises from tightly interconnected interactions among endothelial cells, pericytes, astrocytes, microglia, and infiltrating immune populations [34,68]. Aging-associated microglial priming renders these cells hyperresponsive, fostering excessive cytokine release and perpetuating neuroinflammatory signaling that destabilizes endothelial junctions and compromises blood–brain barrier (BBB) integrity [34,69]. This inflammatory microenvironment transforms the BBB from a selectively protective interface into a structurally vulnerable and functionally compromised barrier [64].

Sustained exposure to inflammatory cytokines disrupts tight-junction organization, alters endothelial signaling pathways, and increases BBB permeability, facilitating penetration of plasma-derived components such as fibrinogen and immune mediators that further amplify neuroinflammation and microvascular dysfunction [40,68]. In parallel, pericytes undergo contraction, apoptosis, and detachment in response to inflammatory stimuli, contributing to impaired capillary regulation, structural instability, and microvascular rarefaction [19,70,71]. These alterations compromise cerebral perfusion and accentuate vulnerability to ischemic and neurodegenerative injury.

A central mechanism linking inflammation to microvascular failure is the augmentation of endothelial–leukocyte interactions. Upregulation of adhesion molecules and pro-inflammatory signaling promotes leukocyte rolling, adhesion, and transmigration, impairing microcirculatory flow and inducing leukostasis [16,50]. This process disrupts perfusion dynamics, amplifies oxidative stress, and perpetuates endothelial injury. Simultaneously, inflammatory signaling increases platelet hyperreactivity and tissue factor expression, fostering a pro-thrombotic environment conducive to microvascular occlusion and fibrin deposition in both peripheral and cerebral beds [50,70,72].

The senescence-associated secretory phenotype (SASP) represents a pivotal molecular bridge between chronic inflammation and vascular aging. Senescent endothelial cells, pericytes, and glial populations secrete pro-inflammatory cytokines, matrix-degrading enzymes, and growth factors that sustain immune activation and accelerate structural degradation of the microvasculature [48,54,55,56,57]. This senescence–inflammation axis provides a mechanistic foundation for the concept of inflammaging as a driver of systemic microvascular decline.

Hemodynamic disturbances further exacerbate this inflammatory environment. Altered shear stress and arterial stiffening promote pro-inflammatory gene expression via mechanosensitive signaling pathways, while aging-associated impairment of shear-dependent anti-inflammatory responses amplifies endothelial dysfunction and chronic immune activation [46,73]. This dynamic interplay between mechanical forces and persistent inflammation magnifies microvascular vulnerability across both peripheral and cerebral domains.

Recent evidence demonstrates that the relationship between peripheral vascular pathology and cerebral microvascular impairment is not unidirectional but instead reflects a bidirectional immunovascular axis in which systemic and neurovascular inflammatory processes continuously reinforce one another. In peripheral artery disease (PAD), chronic production of circulating cytokines, chemokines, and oxidative mediators—such as IL-6, TNF-α, IL-1β, oxidized lipoproteins, and NOX-derived ROS—promotes endothelial dysfunction and propagates systemic vascular injury, ultimately compromising cerebrovascular reactivity and blood–brain barrier (BBB) stability [1,3,9,16,22,23,52,53]. However, neuroinflammation and BBB disruption also exert distal effects on peripheral vessels: microglial activation, astrocytic reactivity, and endothelial senescence facilitate the systemic release of pro-inflammatory cytokines, danger-associated molecular patterns, and endothelial microparticles that circulate back to the periphery, amplifying NF-κB signaling, leukocyte activation, and redox imbalance [33,34,40,48,54,55,56,57,64,68]. Thus, once BBB permeability is compromised, the CNS becomes an active contributor to systemic inflammatory load rather than a passive target, generating a feedback loop wherein PAD-driven systemic inflammation accelerates cerebral microvascular damage, and cerebrovascular inflammation in turn exacerbates peripheral endothelial dysfunction [12,17,25,28,33,34,35,58]. This bidirectional framework supports the concept of a unified neurovascular–peripheral inflammatory circuit in which oxidative stress, chronic inflammation, and cellular senescence interconnect across organ systems to sustain progressive microvascular decline [9,12,17,22,48,54].

Collectively, chronic inflammation generates a pathological milieu in which endothelial dysfunction, oxidative stress, thrombosis, and immune dysregulation converge to dismantle microvascular integrity [17,53,74]. This integrated framework explains the parallel progression of peripheral and cerebral microvascular injury observed in aging and chronic vascular disease, positioning chronic inflammation as a central architect of systemic vascular deterioration and neurovascular fragility [17,34].

## 5. Endothelial Senescence and Vascular Decline

Endothelial senescence represents a pivotal inflection point in the trajectory of microvascular aging, constituting a core mechanism of vasculosenescence and systemic vascular decline [43,48,49,54,75]. While oxidative stress and chronic inflammation introduce cumulative fluctuations in endothelial homeostasis, senescence transforms these recurrent insults into stable, self-perpetuating structural and functional deficits [17,49,56]. Senescent endothelial cells undergo profound epigenetic and metabolic reprogramming affecting mitochondrial dynamics, gene expression profiles, intercellular communication, and secretory behavior, giving rise to a dysfunctional, pro-inflammatory endothelium with diminished reparative capacity and heightened susceptibility to barrier failure and thrombogenesis [49,56,57].

In the peripheral microcirculation—particularly in the context of PAD—endothelial senescence emerges as a direct response to sustained ischemia, oxidative imbalance, and chronic cytokine exposure [22,28,35]. Persistent hypoxia induces mitochondrial dysfunction, DNA damage, and activation of stress-responsive pathways such as p38 MAPK and JNK, converging on the upregulation of cyclin-dependent kinase inhibitors p16^INK4a and p21, thereby driving irreversible cell-cycle arrest [21,54]. This senescent phenotype is characterized by reduced nitric oxide bioavailability, cytoskeletal disruption, impaired antioxidant defenses, and heightened vulnerability to apoptosis, ultimately restricting angiogenic repair and diminishing vascular plasticity [35,43].

Although ischemia is an important contributor to cerebrovascular aging, it is not the only driver of endothelial senescence in the aging brain. Neurodegenerative proteins such as amyloid-β and hyperphosphorylated tau exert direct toxic effects on cerebrovascular endothelial cells, promoting mitochondrial depolarization, excessive ROS production, and mitochondrial permeability transition, ultimately triggering premature senescence and loss of endothelial specialization [33,34,35,58]. Amyloid-β additionally disrupts tight-junction stability, increases intracellular calcium, and activates NF-κB–dependent inflammatory pathways, further accelerating oxidative injury and senescence-associated endothelial dysfunction [33,35]. Beyond proteotoxic stress, age-related arterial stiffness imposes abnormal pulsatile mechanical forces on intracerebral microvessels, generating cyclical shear stress fluctuations and microtrauma to the endothelium [46,47,49,73]. This heightened mechanical load contributes to glycocalyx degradation, junctional disassembly, impaired NO signaling, and activation of mechanosensitive inflammatory cascades that reinforce senescence and microvascular rarefaction. These non-ischemic mechanisms—proteopathic toxicity and stiffness-induced mechanical stress—act synergistically with hypoperfusion to accelerate microvascular decline, BBB vulnerability, and neurovascular unit instability in the aging brain.

A defining biological hallmark of senescent endothelial cells is the senescence-associated secretory phenotype (SASP), marked by the sustained release of pro-inflammatory cytokines, chemokines, growth factors, and matrix metalloproteinases [56,57]. Within PAD, SASP exacerbates leukocyte recruitment, extracellular matrix degradation, and oxidative stress, reinforcing microvascular fragility and promoting systemic dissemination of inflammatory mediators that extend beyond the limb circulation to affect remote vascular beds, including the cerebral microvasculature [56,76,77,78].

Within the cerebral microcirculation, endothelial senescence profoundly undermines BBB integrity through mitochondrial failure, telomere attrition, and genomic instability [36,79,80]. Accumulation of senescent endothelial cells disrupts the tight-junction architecture essential for BBB function, impairing neurovascular coupling and metabolic homeostasis [33,36]. Downregulation and mislocalization of claudin-5 and occludin increase paracellular permeability, enabling extravasation of fibrinogen and albumin into the neural parenchyma, which in turn triggers reactive astrocytosis and microglial activation, further perpetuating neuroinflammation and vascular instability [40,81].

Senescence also disrupts endothelial–pericyte signaling. Reduced PDGF-B expression from senescent endothelial cells weakens pericyte attachment and survival, leading to pericyte loss, capillary constriction, basement membrane thickening, and microvascular rarefaction across both peripheral and cerebral vascular networks [19,20,82]. This destabilization compromises vessel tone regulation and further accentuates microvascular fragility.

Functionally, senescent endothelial cells exhibit impaired vasodilatory capacity due to reduced eNOS activity and diminished nitric oxide production [30,43], aggravating ischemia in PAD and compromising neurovascular responsiveness in the aging brain [30,65]. Concurrently, these cells acquire a pro-thrombotic phenotype characterized by increased expression of tissue factor, von Willebrand factor, and PAI-1, fostering microthrombi formation and contributing to silent microinfarction and subclinical ischemic damage [37,50].

Aging further amplifies these pathological mechanisms through cumulative oxidative and inflammatory stress, reinforcing a vicious cycle between senescence and inflammaging [17,43,62]. SASP-mediated paracrine signaling extends the senescent phenotype to neighboring endothelial populations, propagating dysfunction across vascular territories and accelerating systemic microvascular decline [56,57].

From a therapeutic perspective, emerging strategies targeting endothelial senescence—including senolytics, senomorphics, mitochondrial restoration approaches, and stabilization of endothelial–pericyte interactions—hold promise for mitigating microvascular degeneration and restoring vascular resilience [55,83,84]. By selectively eliminating or functionally reprogramming senescent cells, these interventions may interrupt the self-amplifying cycle of vascular aging and restore microvascular homeostasis [35,77,85].

In summary, endothelial senescence constitutes a central driver of microvascular pathology, integrating oxidative stress, chronic inflammation, BBB disruption, impaired perfusion, and pro-thrombotic transformation into a unified mechanism underlying systemic microvascular aging and neurovascular vulnerability [20,29,30,36,37,38,43,48,49].

## 6. Blood–Brain Barrier Dysfunction as a Convergence Point

**Figure 2:**
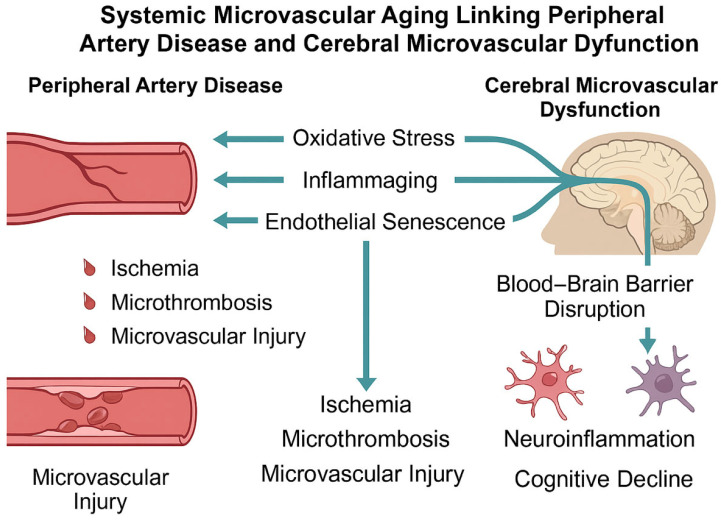
Systemic microvascular aging as a bridge between peripheral artery disease and blood–brain barrier disruption.

The blood–brain barrier (BBB) represents one of the most specialized vascular interfaces in the human body, composed of a highly differentiated endothelial layer supported by pericytes, astrocytic endfeet, and the basement membrane, forming the structural backbone of the neurovascular unit and ensuring strict control over molecular exchange between blood and neural tissue [47,65,76,86]. This sophisticated architecture preserves cerebral homeostasis and neuronal function; however, it also renders the BBB exquisitely vulnerable to systemic insults such as oxidative stress, chronic inflammation, and endothelial senescence [24,87]. Within the continuum of peripheral artery disease (PAD) and systemic vascular aging, the BBB emerges as a critical convergence point where peripheral microvascular pathology translates into cerebral vulnerability [24,25]. The shared molecular and vascular mechanisms linking peripheral artery disease and cerebral microvascular dysfunction are schematically illustrated in Figure 2.

At the core of BBB integrity lies the cerebral endothelium, which is distinguished by dense tight junctions, reduced transcytosis, and a strong reliance on mitochondrial bioenergetics to sustain its high metabolic demands [21,65]. Chronic oxidative and inflammatory stress—whether locally generated or systemically propagated—induces mitochondrial dysfunction, DNA damage, and cytoskeletal disorganization, leading to progressive destabilization of junctional proteins such as claudin-5 and occludin, ultimately compromising paracellular sealing [58,87]. These alterations permit plasma-derived components to penetrate the perivascular and parenchymal spaces, initiating immunovascular injury and amplifying neuroinflammatory cascades [58,88].

This diagram illustrates how systemic microvascular aging links peripheral artery disease (PAD) to cerebral microvascular dysfunction through convergent mechanisms including oxidative stress, inflammaging, and endothelial senescence. These processes promote microvascular injury and ischemia in the periphery, while simultaneously driving blood–brain barrier disruption, neuroinflammation, and progressive cognitive decline within the central nervous system.

BBB disruption develops as a progressive and heterogeneous process, characterized by focal increases in permeability, altered transporter expression, and regional variability in barrier performance [58]. Experimental evidence demonstrates that oxidative stress redistributes claudin-5 and disrupts tight-junction continuity, creating microdiscontinuities that expand under sustained inflammatory pressure [33,89]. As a consequence, plasma proteins such as fibrinogen, albumin, and complement infiltrate the neural parenchyma, triggering cellular stress responses and tissue damage [60,62,90]. Fibrinogen promotes microglial activation and perivascular inflammation [91], albumin induces astrogliosis and cytoskeletal remodeling [92], while complement dysregulation contributes to synaptic dysfunction and neuronal stress responses [60].

In the context of PAD, BBB vulnerability acquires particular significance. PAD generates a chronic systemic environment marked by elevated cytokines, endothelial microparticles, and oxidized lipids [51], all of which dynamically interact with cerebral endothelial cells, impair cerebrovascular reactivity, and increase BBB permeability [24,25]. These alterations contribute to white matter hypoperfusion and subtle BBB leakage, often preceding the clinical onset of cognitive decline in PAD patients [25].

Mitochondrial integrity constitutes a decisive determinant of BBB resilience. Cerebral endothelial cells contain high mitochondrial density to support tight-junction maintenance and energy-intensive barrier regulation [21]. Mitochondrial dysfunction driven by oxidative imbalance, inflammatory signaling, or cellular senescence reduces ATP availability, disrupts cytoskeletal organization, and promotes excessive ROS production, further aggravating tight-junction disassembly and endothelial fragility [58,88].

Pericytes represent another fundamental pillar of BBB stability. These mural cells regulate capillary tone, endothelial survival, and barrier assembly [47]. Chronic oxidative stress and inflammation induce pericyte contraction, detachment, and apoptosis, destabilizing the basement membrane and amplifying vascular permeability [20,25]. Progressive pericyte loss therefore acts as a crucial mechanistic bridge linking systemic vascular pathology to BBB breakdown.

Astrocytic endfeet, which envelop nearly the entire cerebral microvascular surface, play an active role in maintaining BBB integrity through metabolic support and trophic signaling. Under chronic inflammatory and oxidative conditions, astrocytes undergo reactive transformation, characterized by GFAP overexpression, increased MMP secretion, and diminished angiopoietin-1 signaling, all of which weaken endothelial junctions and basement membrane stability [27,92]. In parallel, BBB leakage triggers microglial activation, shifting these cells toward a pro-inflammatory phenotype that releases cytokines and ROS, perpetuating a self-reinforcing cycle of endothelial injury and neuroinflammation [91,93].

Hemodynamic disturbances further exacerbate BBB instability. Arterial stiffness, reduced elasticity, and impaired autoregulation generate abnormal pulsatile forces that disrupt endothelial junctions and basement membrane architecture [61,94]. These mechanical stressors synergize with biochemical insults to promote microbleeds, capillary rarefaction, and perivascular fibrosis, accelerating structural and functional BBB decline.

Collectively, BBB dysfunction represents the intersection of oxidative stress, chronic inflammation, endothelial senescence, mitochondrial injury, pericyte loss, astrocytic reactivity, and hemodynamic stress [24,33,58,60,88,94]. Clinically, increased BBB permeability correlates with cerebral small-vessel disease, white matter damage, cognitive decline, neurodegeneration, and elevated stroke risk, positioning BBB integrity as both a mediator and early biomarker of systemic microvascular failure [63,78,95]. These clinical associations closely mirror those described in Alzheimer’s disease, where chronic BBB breakdown, microvascular rarefaction, and neurovascular unit disruption facilitate amyloid-β accumulation, tau pathology, and progressive cognitive decline [58,96].

In summary, BBB disruption constitutes the cerebral manifestation of systemic vascular aging and PAD-associated microvascular pathology. Its progressive breakdown not only reflects the interconnectedness of peripheral and cerebral vascular beds but also reframes microvascular disease as a unified pathophysiological continuum susceptible to shared therapeutic strategies targeting oxidative stress, inflammation, senescence, and endothelial instability [1,3,22,24,25,28,33,35,49,51,58].

## 7. Therapeutic Strategies Targeting the Microcirculation

### 7.1. Molecular and Cellular Modulation of Microvascular Dysfunction

Restoring microvascular integrity requires direct intervention on the fundamental molecular drivers of endothelial injury, namely oxidative stress, chronic inflammation, mitochondrial dysfunction, and cellular senescence. Mitochondria-targeted antioxidants such as elamipretide and other mitochondria-penetrating compounds offer superior efficacy compared to classical antioxidants by attenuating reactive oxygen species at their source, stabilizing mitochondrial membranes, and preserving endothelial tight junction architecture [21,92,97,98,99]. These strategies are particularly relevant in PAD and aging cerebral vasculature, where mitochondrial dysfunction plays a central pathogenic role [21,31].

Targeting NADPH oxidase activity represents another crucial approach, as NOX2 and NOX4 contribute significantly to endothelial oxidative burden and barrier disruption. Selective NOX inhibition has demonstrated protective effects on endothelial integrity, reduced inflammatory activation, and stabilization of microvascular architecture in experimental models [27,91,93]. Complementary anti-inflammatory interventions, including IL-1β blockade and broader inflammasome inhibition, further reduce endothelial activation, leukocyte adhesion, and BBB permeability, reinforcing microvascular resilience [63,78,94,95].

Addressing endothelial senescence has emerged as a transformative strategy. Senolytic agents eliminate senescent endothelial populations, while senomorphic compounds such as metformin, rapamycin, and sirtuin modulators suppress SASP expression and restore vascular homeostasis [84,100,101,102]. These therapies reduce fibrosis, improve endothelial nitric oxide production, and partially reverse microvascular aging, making them particularly relevant for PAD-associated and age-related cerebral microvascular decline [29,54,55].

NAD^+^ precursors and sirtuin activators represent a central metabolic axis for restoring endothelial resilience in both peripheral and cerebral microvascular beds. Declining NAD^+^ levels—accentuated by oxidative stress, chronic inflammation, and mitochondrial dysfunction—impair the activity of sirtuins (SIRT1, SIRT3), key regulators of mitochondrial biogenesis, endothelial nitric oxide synthase (eNOS) stability, antioxidant defense, and endothelial longevity [21,44,48,84,100,101,102,103,104]. NAD^+^ precursors such as nicotinamide riboside and nicotinamide mononucleotide enhance SIRT1-PGC-1α signaling, promoting mitochondrial biogenesis, improving ATP production, and reducing ROS generation in endothelial cells [21,84,103]. In parallel, sirtuin activation suppresses pro-inflammatory transcriptional programs, limits NF-κB activity, attenuates SASP secretion, and stabilizes endothelial tight junctions—mechanisms particularly relevant to PAD-associated microvascular dysfunction and BBB vulnerability [30,49,54,55,65,84,100,101,102].

At the cerebrovascular level, SIRT3-dependent mitochondrial regulation improves redox homeostasis and prevents oxidative uncoupling of eNOS, while SIRT1 preserves claudin-5 expression and endothelial metabolic efficiency, thereby mitigating BBB permeability and neurovascular uncoupling [21,30,36,65,104]. Importantly, aerobic exercise serves as a physiological enhancer of this NAD^+^–sirtuin axis, increasing endogenous NAD^+^ synthesis, stimulating AMPK-SIRT1-PGC-1α activation, improving endothelial NO bioavailability, and promoting angiogenesis and neurovascular coupling in aging individuals [105,106,107]. Through these combined metabolic effects, NAD^+^ augmentation, sirtuin activation, and exercise synergistically counteract oxidative injury, endothelial senescence, and microvascular rarefaction, strengthening the molecular foundation for systemic and cerebral microvascular repair in vascular aging and PAD [1,25,49,51,103,104].

### 7.2. Neurovascular Unit Repair and Structural Reinforcement

Beyond endothelial cells, preserving the integrity of the neurovascular unit is essential for sustained microvascular recovery. Pericytes, astrocytes, and basement membrane components play central roles in maintaining barrier stability and capillary function. Strategies aimed at reinforcing endothelial–pericyte communication via angiopoietin-1/Tie2 signaling and PDGF-B modulation enhance microvascular stability and counteract capillary rarefaction [82,108].

Astrocytic regulation represents an additional therapeutic target. Modulating astrocytic reactivity and preserving AQP4 polarization helps maintain BBB homeostasis and extracellular fluid balance, limiting neuroinflammatory amplification [36,37,38]. Simultaneously, interventions aimed at reducing microglial overactivation through CSF1R inhibition and inflammasome modulation attenuate chronic neuroinflammation and secondary endothelial injury [63,89,95,109].

Matrix metalloproteinase inhibition provides structural stabilization by preventing basement membrane degradation and preserving endothelial junction cohesion, particularly in the context of ischemia and chronic inflammation [110]. Together, these strategies reinforce the structural framework of the microvasculature, supporting both cerebrovascular resilience and peripheral tissue perfusion.

### 7.3. Systemic, Metabolic and Translational Interventions

Given that microvascular dysfunction is systemic in nature, therapeutic intervention must extend beyond local endothelial repair. Lifestyle-based strategies remain foundational. Aerobic exercise enhances endothelial nitric oxide bioavailability, improves cerebral perfusion, promotes angiogenesis, and strengthens neurovascular coupling [105,106,107]. Statins exert pleiotropic endothelial-protective effects independent of lipid lowering, reducing oxidative stress and inflammatory signaling pathways [61,62].

Hemodynamic optimization also plays a critical role. Control of arterial stiffness and hypertension through ACE inhibitors and ARBs contributes to BBB preservation and improved cerebral autoregulation, mitigating mechanical stress on fragile microvessels [111,112]. In PAD, revascularization strategies restore distal shear stress and promote endothelial recovery, indirectly benefiting systemic and cerebral microcirculatory health [113].

Emerging translational interventions, including nanoparticle-mediated drug delivery systems, enhance targeted delivery to endothelial and perivascular cells, improving therapeutic precision while minimizing systemic toxicity [114,115,116]. Metabolic therapies supporting mitochondrial biogenesis and enhancing cellular energy efficiency further reinforce endothelial resilience, delaying progression of microvascular aging and cognitive decline [103,104,117,118,119].

#### Limitations of Standard Vascular Therapies in Microvascular Aging

Although standard vascular therapies—such as statins, ACE inhibitors, and ARBs—provide important macrovascular benefits, their ability to halt microvascular aging is limited. Statins and RAAS-modulating drugs reduce oxidative stress and inflammation but do not directly target mitochondrial dysfunction, DNA damage, endothelial senescence, or neurovascular-unit degeneration, which constitute the core drivers of microvascular decline [43,48,49]. Furthermore, these therapies have modest effects on reversing endothelial SASP, pericyte loss, or BBB instability, all of which progress despite adequate control of systemic risk factors [30,35,65]. As a result, conventional treatments slow but do not reverse the progressive deterioration of microvascular structure and function, highlighting the need for senolytics, NAD^+^ boosters, mitochondrial stabilizers, and neurovascular-unit–targeted therapies that more directly address the mechanisms of vascular aging [55,83,84,103,104].

### 7.4. Integrated Therapeutic Perspective

From a translational standpoint, most of the therapeutic strategies discussed in this review remain at an early stage of clinical development. Mitochondria-targeted antioxidants and NOX inhibitors have shown consistent protective effects on endothelial function, redox balance, and microvascular architecture in experimental models of cardiovascular and neurovascular injury, but only a limited number of compounds have advanced to small phase I–II trials with heterogeneous effects on clinical endpoints [21,27,91,92,93,97,98,99]. Senolytic agents, including dasatinib–quercetin combinations and other senescence-targeting drugs, have demonstrated robust vasculoprotective and anti-inflammatory actions in preclinical models and early human studies; however, long-term data on cerebrovascular outcomes, cognitive trajectories, and BBB integrity are still lacking [55,83,84]. Similarly, NAD^+^ precursors and sirtuin activators such as nicotinamide riboside, nicotinamide mononucleotide, and metformin have been associated with improved mitochondrial function, endothelial reactivity, and cardiometabolic profiles in pilot clinical trials, yet their capacity to reverse established microvascular aging or prevent neurovascular decline remains to be demonstrated [21,84,100,101,102,103,104]. Overall, these interventions should therefore be regarded as promising, mechanistically grounded candidates rather than established therapies, underscoring the urgent need for large, long-term randomized trials with vascular and cognitive outcomes in patients with PAD and age-related cerebrovascular disease.

Recent advances in translational vascular medicine have expanded the therapeutic landscape; however, the clinical development of these strategies remains heterogeneous and incompletely validated. Mitochondria-targeted antioxidants such as elamipretide (SS-31) have progressed into phase II clinical trials, showing improvements in mitochondrial bioenergetics and vascular function but producing mixed results regarding long-term functional outcomes [21,92,97,98,99]. Selective NOX inhibitors, including GKT137831, have completed phase I/II trials with favorable safety profiles and modest reductions in inflammatory biomarkers, though no phase III data are available to confirm microvascular benefit [27,91,93].

Senolytic agents—particularly dasatinib–quercetin combinations—have demonstrated strong preclinical vasculoprotective effects and have reached early-phase human studies, where they reduced systemic inflammation and improved physical function; nevertheless, their effects on cerebrovascular integrity, cognitive outcomes, and BBB stability remain untested in large clinical cohorts [55,83,84].

NAD^+^ boosters such as nicotinamide riboside and nicotinamide mononucleotide have been evaluated in phase I/II trials, showing enhanced mitochondrial function and endothelial responsiveness, yet no evidence currently supports reversal of established microvascular aging or prevention of neurovascular decline [21,84,100,101,102,103,104].

Overall, most interventions discussed in this review remain in early translational stages, with limited progression to controlled randomized trials. Large, long-term studies with vascular and cognitive endpoints are urgently required to determine their therapeutic relevance in PAD-associated and age-related cerebral microvascular disease.

Ultimately, microvascular restoration requires a multidimensional approach that integrates molecular repair, structural stabilization, and systemic optimization. Interventions targeting oxidative stress, inflammation, senescence, mitochondrial dysfunction, and neurovascular unit degradation operate synergistically to stabilize the microcirculatory network and protect both peripheral and cerebral tissues [1,25,49,51]. This unified strategy reframes microvascular disease not as a localized pathology but as a systemic continuum amenable to coordinated therapeutic modulation.

## 8. Future Perspectives

**Figure 3:**
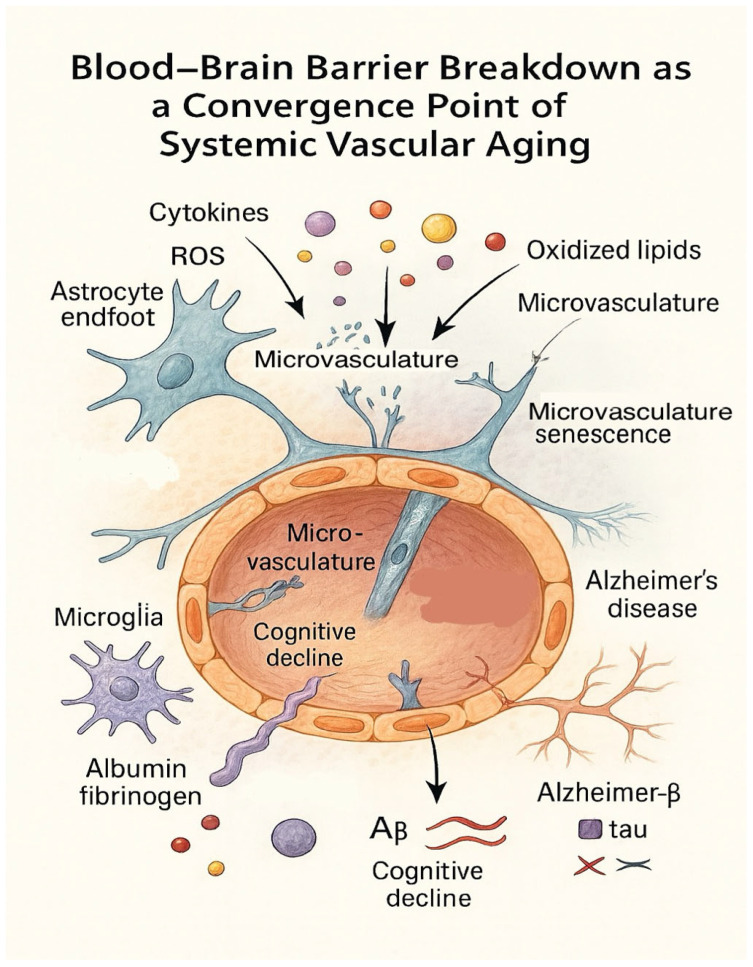
Blood–brain barrier breakdown as a convergence point of systemic vascular aging and Alzheimer’s disease.

The recognition of microvascular decline as a systemic and interconnected process has opened new horizons for predictive diagnostics and integrative therapeutic frameworks. Rather than conceptualizing peripheral artery disease (PAD) and cerebral microvascular dysfunction as independent entities, growing evidence supports a unified vascular paradigm in which shared molecular drivers-oxidative stress, chronic inflammation, mitochondrial dysfunction, endothelial senescence, and neurovascular unit destabilization—define both disease trajectory and therapeutic responsiveness [1,3,25,49,51]. The integrated molecular and cellular pathways discussed in this section are schematically summarized in Figure 3.

As populations age and the prevalence of vascular cognitive impairment, cerebral small-vessel disease, and PAD continues to rise, the capacity to detect early microvascular alterations and initiate intervention before irreversible structural damage occurs has become a central priority in vascular and neurodegenerative medicine [9,25,51].

### 8.1. Precision Diagnostics and Early Detection of Microvascular Injury

Future advances in microvascular medicine will increasingly depend on the identification of subclinical endothelial dysfunction through highly sensitive molecular and imaging platforms. Circulating endothelial microparticles, inflammatory mediators, oxidative stress markers, and endothelial-derived extracellular vesicles have emerged as promising indicators of systemic vascular stress and early endothelial injury [10,28,50,62]. These biomarkers provide dynamic insight into endothelial phenotype, inflammatory burden, and microvascular remodeling long before overt clinical manifestations develop.

Advanced neuroimaging techniques—including dynamic contrast-enhanced MRI and arterial spin labeling—now enable in vivo visualization of BBB permeability, perfusion heterogeneity, and subtle neurovascular dysfunction, facilitating the detection of microvascular injury at preclinical stages [26,120,121]. When combined with circulating biomarkers, these technologies offer a multidimensional assessment of vascular integrity and neurovascular coupling, enhancing risk stratification accuracy and early therapeutic decision-making [24,122].

Emerging transcriptomic and proteomic profiling of endothelial and pericyte-derived extracellular vesicles may further refine predictive diagnostics by identifying molecular signatures of mitochondrial dysfunction, endothelial senescence, and BBB vulnerability [30,54,80,98]. Such approaches hold promise in defining individualized microvascular phenotypes and guiding precision therapeutic targeting.

### 8.2. Regenerative, Molecular and Targeted Therapeutic Innovations

Future therapeutic strategies will increasingly emphasize restoration and regeneration rather than symptomatic compensation. Regenerative approaches using endothelial progenitor cells and iPSC-derived vascular cells demonstrate potential for reconstructing damaged microvascular networks in both PAD and cerebral small-vessel disease, promoting revascularization and functional recovery [82,123,124].

Nanotechnology-based delivery systems offer highly targeted transport of antioxidants, senolytics, mitochondrial stabilizers, and anti-inflammatory agents directly to endothelial or perivascular compartments, improving therapeutic efficacy while minimizing systemic toxicity [11,115,116]. Intranasal delivery platforms further enhance central nervous system penetration, facilitating direct modulation of neurovascular unit components and BBB repair [125].

At the molecular level, modulation of mitochondrial metabolism through NAD^+^ boosters and metabolic reprogramming strategies may strengthen endothelial resilience and delay vascular aging progression [31,103,104]. These approaches reinforce the concept of treating the neurovascular unit as an integrated therapeutic target rather than isolated cellular elements [35,74].

### 8.3. Integrated Vascular Medicine and Predictive Systems

The future of microvascular care will likely involve unified clinical models that address peripheral and cerebral circulation simultaneously. Artificial intelligence–driven computational modeling and systems biology approaches are positioned to integrate clinical, imaging, and molecular data, enabling predictive identification of microvascular decline and personalized therapeutic stratification [126].

Lifestyle-based modification remains fundamental. Aerobic physical activity, metabolic optimization, and structured vascular rehabilitation programs demonstrate sustained improvement in endothelial function, cerebral perfusion, and neurovascular coupling [105,106,107]. These strategies complement pharmacological and regenerative approaches by preserving long-term vascular adaptability and resilience.

Ultimately, the convergence of precision diagnostics, regenerative therapeutics, targeted drug delivery, and systems-based predictive models will redefine microvascular medicine as a proactive, anticipatory, and integrative discipline rather than a reactive one constrained to late-stage intervention [1,25,49,51].

This conceptual illustration depicts how systemic vascular aging promotes microvascular senescence and dysfunction, leading to progressive disruption of the blood–brain barrier (BBB). Increased oxidative stress, pro-inflammatory cytokines, and oxidized lipids compromise endothelial integrity and astrocytic endfoot interactions, facilitating the extravasation of plasma proteins such as albumin and fibrinogen. These alterations contribute to neuroinflammation, microglial activation, and the accumulation of amyloid-β (Aβ) and tau pathology, ultimately driving cognitive decline and the progression of Alzheimer’s disease. The figure highlights the central role of the cerebral microvasculature as an integrative axis linking systemic vascular deterioration with neurodegenerative processes.

## 9. Conclusions

This review consolidates current evidence supporting microvascular dysfunction as a unified and systemic process linking peripheral artery disease (PAD) and cerebral microvascular pathology, rather than two independent and compartmentalized entities. Across both vascular territories, convergent mechanisms—oxidative stress, chronic inflammation, mitochondrial dysfunction, endothelial senescence, and destabilization of the neurovascular unit—orchestrate progressive structural and functional deterioration that ultimately compromises tissue perfusion, barrier integrity, and metabolic resilience [1,3,8,25,49,51,87].

The microvasculature emerges not as a passive conduit but as a dynamic regulatory interface whose dysfunction precedes overt clinical manifestations. In PAD, microvascular injury accelerates ischemia, endothelial failure, and inflammatory amplification, whereas in the brain it promotes blood–brain barrier (BBB) disruption, neuroinflammation, impaired neurovascular coupling, and progressive cognitive decline [24,25,28,33,35]. These shared pathological drivers strongly support a paradigm shift from organ-specific models toward a systemic continuum of microvascular disease.

Endothelial senescence represents a pivotal inflection point in this trajectory, transforming potentially reversible endothelial dysfunction into irreversible vascular decline through SASP-mediated inflammatory perpetuation, impaired regenerative capacity, and enhanced thrombogenicity [12,48,49,54,55,56,57,80]. Simultaneously, mitochondrial dysfunction not only fuels oxidative imbalance but destabilizes endothelial signaling and BBB regulation, reinforcing a self-sustaining cycle of progressive microvascular damage [21,30,31].

The BBB emerges as a highly sensitive integrator of systemic vascular stress. Its deterioration reflects both intrinsic neurovascular injury and remote peripheral vascular disturbances, positioning BBB disruption as an early biomarker and therapeutic target in systemic vascular aging [24,32,33,68,88]. This reinforces the concept that cerebral microvascular compromise is not an isolated neurological phenomenon but an extension of systemic vascular pathology.

Therapeutic strategies must therefore evolve beyond symptom-oriented interventions. Integrated approaches targeting oxidative stress, inflammatory signaling, endothelial senescence, mitochondrial metabolism, and neurovascular unit integrity offer the greatest potential for stabilizing microvascular architecture across both peripheral and cerebral domains [42,44,84,100,101,102,103,104,105,108,110,127]. In parallel, regenerative medicine, nanotechnology-based delivery systems, and precision therapeutic models hold promise for restoring microvascular homeostasis in vulnerable populations and delaying disease progression.

Ultimately, recognizing PAD and cerebral microvascular pathology as interconnected manifestations of systemic vascular aging reframes clinical management within a unified vascular framework. This shift supports early detection, predictive diagnostics, and coordinated therapeutic strategies aimed at preserving cognitive function, reducing ischemic risk, and prolonging microvascular integrity throughout aging [49,51,87].

By advancing an integrated systems-based interpretation of microvascular pathology, this review supports a paradigm shift in which cerebral and peripheral vascular diseases are no longer treated as compartmentalized conditions, but as interconnected expressions of a common vascular aging continuum. This conceptual repositioning opens the door to earlier diagnostic strategies and unified therapeutic targets aimed at preserving endothelial resilience and neurovascular integrity across the lifespan [1,25,49,51,78,95].

## Figures and Tables

**Table 1:** Circulating biomarkers shared between peripheral artery disease (PAD) and cerebral microvascular dysfunction.

Biomarker Category	PAD Evidence	Cerebral Microvascular Evidence	Pathophysiological Relevance
Inflammatory cytokines (IL-6, TNF-α, IL-1β)	Elevated in symptomatic PAD; associated with endothelial dysfunction [18,22,52]	Increased in cerebral small-vessel disease and BBB disruption [34,63]	Activate NF-κB, promote leukocyte adhesion, amplify oxidative stress
Oxidative stress markers (ox-LDL, MDA, protein carbonyls)	Robustly elevated in PAD plasma [1,3]	Increased in vascular cognitive impairment and AD [33,35]	Drive lipid peroxidation, mitochondrial injury, endothelial instability
Endothelial microparticles (EMPs)	Increased EMPs indicate endothelial apoptosis and vascular injury [23,53]	EMPs correlate with BBB leakage and microvascular dysfunction [33,64]	Reflect systemic vascular stress, senescence, endothelial detachment
SASP factors (MMP-2, MMP-9, PAI-1)	Elevated in chronic ischemia and PAD-related inflammation [54,55]	Increased in aging brain and CSVD [48,57]	Mediate matrix degradation, inflammation, and vascular remodeling
Nitric oxide metabolism markers (ADMA, SDMA)	ADMA elevated in PAD; predicts endothelial dysfunction [16,18]	Elevated in impaired neurovascular coupling [65]	Inhibit eNOS, reduce NO, worsen microvascular function
Endothelial glycocalyx markers (syndecan-1, heparan sulfate fragments)	Increased in advanced PAD [23]	Correlate with BBB permeability and microbleeds [33,58]	Indicate structural vascular fragility

## Data Availability

No new data were created or analyzed in this study. Data sharing is not applicable to this article.
